# Binding of Task-Irrelevant Action Features and Auditory Action Effects

**DOI:** 10.5334/joc.225

**Published:** 2022-06-06

**Authors:** Sámuel Varga, Roland Pfister, Bence Neszmélyi, Wilfried Kunde, János Horváth

**Affiliations:** 1 Institute of Cognitive Neuroscience and Psychology, Research Centre for Natural Sciences, Budapest, Hungary; 2 Department of Cognitive Science, Faculty of Natural Sciences, Budapest University of Technology and Economics, Hungary; 3 Department of Psychology, University of Würzburg, Würzburg, Germany; 4 Institute of Psychology, Károli Gáspár University of the Reformed Church in Hungary, Budapest, Hungary

**Keywords:** action control, binding and retrieval, action-effect binding, action parameters, response force

## Abstract

Discrete task-relevant features of an overt response, such as response location, are bound to, and retrieved by coincidentally occurring auditory stimuli. Here we studied whether continuous, task-irrelevant response features like force or response duration also become bound to, and retrieved by such stimuli. In two experiments we asked participants to carry out a pinch which produced a certain auditory effect in a prime part of each trial. In a subsequent probe part, tones served as imperative stimuli which either repeated or changed as compared to the effect tone in the prime. We conjectured that the repetition of tones should result in more similar responses in terms of force output and duration as compared to tone changes. Most parameters did not show notable indications for such similarity increases, including peak force or area under force curve, though the correlation between response durations in prime and probe was higher when tones repeated rather than changed from prime to probe. We discuss these results regarding perceptual discriminability and deployment of attention to different nominally task-irrelevant aspects of pinch responses.

## Introduction

Everyday life is a sequence of interactions with the physical environment. We perform actions with the intent of achieving different *goals* (e.g., turning on the light, launching an application on a smartphone, waving to catch the attention of our friends), without being aware of the complex biomechanical interplay that underlies our movements. We also rarely become aware of how many different movements we could perform to achieve the same desired outcome. Such interactions often lead to other, goal-irrelevant effects in the environment and in our body, which may nonetheless influence action control (Cao et al., 2020; Horváth et al., 2018; Pfister, 2019). The human cognitive system is geared towards using any of these perceptual action effects for decision-making and action planning alike (Wolpert et al., 2011).

According to recent approaches to action control, stimulus and motor features activated during any motor action are integrated (bound) to each other (Frings et al., 2020; Hommel, 2009; 2019). In turn, re-activating a feature leads to the activation (or retrieval) of all associated stimulus and action features.

Binding and retrieval have been characterized as highly automatic processes in that they operate on relevant and irrelevant stimulus features alike (Bogon et. al., 2017; Cochrane & Milliken, 2019; Dutzi & Hommel, 2009; Frings et al., 2007; Hommel, 2005; Moeller et al., 2016). Whether or not the same applies to irrelevant motor features is an open question, however. The everyday observations mentioned above suggest that these features go surprisingly unnoticed even right at the moment of planning and performing an action. This property contrasts with irrelevant stimulus features as commonly operationalized in corresponding experiments, where even irrelevant stimulus features are commonly distinct and readily perceivable. We therefore conducted the present study to investigate whether task-irrelevant force patterns during pinching are bound to, and retrieved by, task-irrelevant tone effects that are triggered by each pinch.

Action-effect binding and retrieval is typically investigated in tasks that pair discrete actions such as keypresses with following tone effects (Dutzi & Hommel, 2009; Herwig & Waszak, 2012; Janczyk et al., 2012; Schwarz et al., 2018). Studies in this literature commonly use prime-probe designs in which binding can occur in the prime part (e.g., by experiencing an action-effect episode) whereas the probe part tests whether potential bindings are actually retrieved upon re-encountering the previous effect as an imperative stimulus (as compared to encountering an alternative stimulus that had not occurred in the prime part). That is: Observing characteristic behavioural changes depending on the repetition or change of a previous response-effect from prime to probe allows inferring that binding and retrieval must have occurred. Behavioural changes can relate to choices if the probe stimulus allows for free choice (testing whether repeating the previous effect increases the participants’ tendency to repeat their prime response), or they can relate to response time differences in forced-choice settings (Moeller et al., 2016, 2019). In the latter case, probe response times are lower when effect/stimulus and response both repeat or both change from prime to probe, as compared to cases in which only either the effect/stimulus or the response repeats.

But what actually gets bound in such action-effect bindings? Each individual action comes with numerous unique features, such as a categorical decision (e.g., “left key”) as well as different body-related effects such as proprioceptive and kinaesthetic changes produced by the actual movement (Pfister, 2019; Pfister et al., 2014). Whether re-encountering a previous effect retrieves categorical features involved in decision-making or whether it retrieves the actual body-related effects is thus an open question. While this distinction applies to any type of binding and retrieval paradigm, action-effect binding might be expected to yield particularly high chances of observing retrieval of actual movements. Typical designs allow for presenting the effect immediately after the response, thus implementing close temporal proximity of response and effect onset. Preliminary evidence on binding and retrieval for action slips further suggests that action-effect binding is particularly sensitive to actually executed as compared to intended but not executed actions, whereas the reverse is true for stimulus-response binding (Foerster et al., 2021). Moreover, even though metric properties of the eventual body movement are nominally task-irrelevant by instruction, they are still important when it comes to optimizing movements (Neszmélyi & Horváth, 2017), and might also become bound to sensory action effects. The particular case of pinching actions, for instance, comes with the metric parameters of peak force, latency of the peak, area under the curve, action duration, acceleration and deceleration etc., each of which might be a good indicator of binding effects in simpler movements. It seems reasonable to treat these action properties similarly, but as we speculate in this study, there could be relevant differences between them, such as perceptibility. Specifically, action force might be less observable than action duration (previous studies suggest that while the kinematics of movements are easily accessible, this is not the case when it comes to muscular forces; de Graaf et al., 2004). Furthermore, while previous results focus on categorical or task-relevant response features in choice response tasks (e.g., Kunde, 2001), it remains to be examined how binding and retrieval plays out when only a single interaction option is available, hence in ‘single’ response task.

We investigated these questions in two experiments using a modified prime-probe design (partly based on the paradigm used by Moeller et al., 2016), with the pinching of a force-sensitive resistor (FSR) being the only response option. As indicated above, while binding effects in choice tasks tend to manifest themselves in reaction times or repetition/alternation tendencies, we hypothesized that these effects might also be detectable in other, task-irrelevant properties of actions. Specifically, we conjectured that actions on the prime and probe would be more similar as a result of tone repetitions (congruent trials) compared to tone switches (incongruent trials). We therefore studied action-effect binding with a special focus on action force, but other movement features, like reaction times and action durations were also inspected.^1^

## Experiment 1

### Method

#### Participants

19 young adult participants took part in the experiment: mean age 21.5 years (range: 19–30, 3 left-handed, 13 women). This sample size allowed us to detect an effect size of *d*_z_ = 0.68 with 1–β = .80 at *α* = .05, whereas action-effect binding has usually been reported to come with substantially larger effects (e.g., *d_z_* = 1.06 in Experiment 1 of Moeller et al., 2016).

Participants reported normal hearing and no diagnosed neurological disorders.

#### Materials, stimuli and data acquisition

The device used in the experiment was a single-zone Force Sensing Resistor (FSR 400, Interlink Electronics, Westlake Village, CA, USA; 0.3 mm thick, active area of 5.1 mm diameter) mounted on a thin plastic sheet. During the experiment, the FSR was held between the index finger and the thumb, with the thumb pressing down on it. The FSR signal was recorded with a voltage-divider setup using the high-level input of a SynAmps2 EEG amplifier (Compumedics Neuroscan, Victoria, Australia). The sampling rate was 1000 Hz, with a 200 Hz online low-pass filter. The FSR signal values were transformed into force values using an exponential transformation.

The auditory stimuli presented as prime and probe (see below) were 440 Hz and 1175 Hz, 60 dB pure sinusoidal tones that were 300 ms long with 5 ms long linear rise and fall times. Tones were delivered via headphones (Sennheiser HD-600, Wedemark, Germany). Because of hardware constraints, there was a constant 6 ms delay in the presentation of the tones in relation to the detected action onsets.

Instructions and feedback were presented on a 24 inch, 1920 × 1080 pixels resolution liquid crystal display (BenQ XL2430) placed approximately 1 m in front of the participant.

The experiment was run by custom scripts in GNU Octave (Eaton et al., 2014) using the Psychophysics Toolbox (Brainard, 1997; Pelli, 1997, Kleiner et al., 2007) on a Linux operating system.

#### Task and procedure

The experiment was conducted in a sound-attenuated room where participants sat in an armchair. At the beginning of the experiment, the experimenter presented the proper way to hold the device, then demonstrated the correct execution of the actions, with the FSR signal curves appearing on a monitor. Participants were encouraged to produce brief pinches during the experiment, then were left to inspect and freely pinch the FSR with visual feedback on the FSR signal. After being familiarized with the device, the following instructions were given: *‘Your task will be to wait for 2 seconds, then press (pinch) the device. The pinch will be followed by a sound, and sometimes by a second one as well. If you hear a second sound, quickly pinch the device! Let us know when you are ready to begin!’* If the participants asked questions, the instructions were clarified, and when ready, participants worked through one practice block containing 20 trials (10 congruent and 10 incongruent trials and no catch trials; see below).

**Figure 1:**
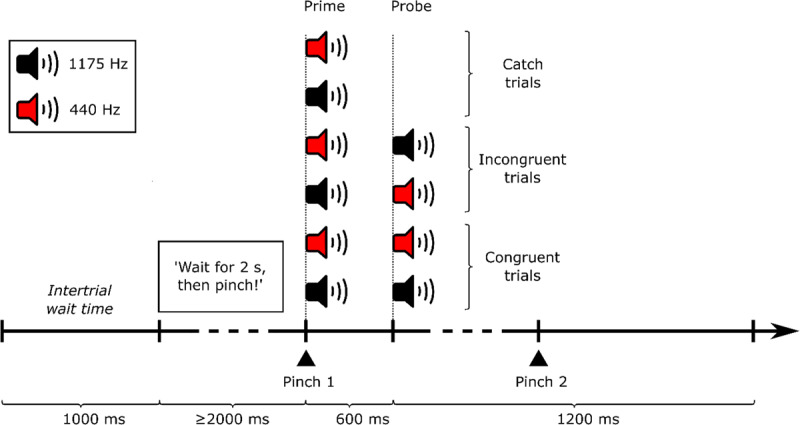
Trial structure in Experiment 1. The prime part of each trial featured a first pinch that immediately triggered either a high or low effect tone. The probe part then either repeated this tone as a stimulus (congruent trials), featured the alternative stimulus (incongruent trial) or did not present any tone (catch trial). Participants were instructed to perform a second pinch whenever they heard a second tone but to refrain from responding in catch trials. The timing represented on the horizontal axis is not to scale.

Figure 1 showcases the trial procedure for all experimental conditions. Each trial began with the following text appearing on the screen: *‘Wait for 2 seconds, then pinch!’* The screen did not change for the duration of the trial, except for trials with too early actions/responses. In case the participant did not wait for at least 2 seconds and pinched too early, the following warning appeared on the screen: *‘Too fast! Wait for 2 seconds!’* The text was present for 1 second, after which the trial was repeated, starting with the wait time of at least 2 s. After registering a successful pinch, a low or high-frequency tone was presented. This effect tone was followed either by the same tone (congruent trials) or a different tone (incongruent trials) after 600 ms or – in the case of catch trials – no second tone was presented. If the participant pinched within the 600 ms following the onset of the first tone, a warning appeared for 1 s: *‘Wait for the sound, please!’* The pinch was registered as a false alarm and the trial ended. If the participant only pinched after the arrival of the second tone (as instructed), the reaction time was recorded. If the participant did not pinch within 1200 ms after the onset of the second tone, the trial was recorded as a missed event. For catch trials, the total time until the end of the trial was 1800 ms (600 ms following the self-induced tone onset plus 1200 ms response interval). The next trial started after an inter-trial interval of 1 s. At the end of each block, a feedback message displayed the number of false alarms and the number of missed events.

Participants completed 7 experimental blocks of 30 trials, with each experimental block consisting of 12 congruent, 12 incongruent and 6 catch trials. Trial order was pseudo-randomized with the constraint that the first two trials of each block could not be catch trials.

#### Data selection and analyses

Trials with reaction times faster than 100 ms (*M* = 1.6% of trials, not including catch trials, *SD* = 2.4%, range = 0.0–8.8%) and late responses with reaction times longer than the mean reaction time + 2.5 standard deviations were rejected from the analysis as outliers (*M* = 1.8%, *SD* = 1.1%, range = 0.6–3.1%; computed per participant and condition). To ensure that force application was finished before the second tone onset, trials with first pinch durations equal to or longer than 600 ms were rejected (*M* = 3.4% of remaining trials, *SD* = 4.6%, range = 0.0–13.0%). The same criterion was applied to probe-related pinches (1.3% of remaining trials, *SD* = 2.1%, range = 0.0–7.3%). After this rejection procedure, the average number of remaining trials per participant was 76 (*SD* = 6.8, range = 56–84) in the congruent condition, and 74 (*SD* = 7.3, range = 60–84) in the incongruent condition.

Force application patterns were characterized by peak force and the temporal integral of force (area under the curve – *AUC*) for each trial. For AUC calculations, the onset and end of each individual pinch were identified. Action onset was registered when force exceeded a pre-set threshold of 0.32 N after being continuously under the threshold for at least 10 ms. The endpoint was registered as the timepoint when the force signal dropped below the threshold and then stayed under it for at least 10 ms. The z-score-based outlier rejection procedure mentioned above was performed also for each dependent variable relating to force and duration, that is, trials with z-scores below or above 2.5, computed separately for each participant and experimental condition were rejected from the analysis.

We compared 1) the intraindividual correlation of prime and probe force across trials between conditions, 2) the mean difference of prime and probe force between tone repetitions and tone switches and 3) reaction times on congruent and incongruent trials. Further, exploratory analyses were performed on action duration. Using the BayesFactor package for R (version 0.9.12–4.2), we also calculated Bayes Factors for the corresponding t-tests of the main analyses, using a Cauchy prior centred at zero with the default scale parameter of .707. We always report the Bayes Factor that shows the evidence for the alternative hypothesis over the null hypothesis (i.e., *BF*_10_).

## Results

### Error rates and misses

There were on average 3.1 missed trials per participant (*SD* = 4.6), representing 1.9% of trials, excluding catch trials (*SD* = 2.7%, range = 0.0–10.0%). The number of catch trial errors was relatively low, representing on average 1.2% of all trials (*SD* = 1.2%, range = 0.0–3.8%) and 6.1% of catch trials (*SD* = 6.0%), but there was considerable between-participant variability (range = 0.0–19.5%). Similarly, there was considerable variance in the number of trial restarts (i.e., when the participant did not wait for at least 2 s before pinching after the start of the trial), with values ranging from 2 to 41 trial restarts per participant (*M* = 17, *SD* = 11). Furthermore, the occurrence of pinches before the second tone arrived was low as well, representing on average 0.8% of all trials (*SD* = 1.2%, range = 0.0–3.4%).

### Force analyses

Pearson’s correlations between prime and probe force (AUC) were calculated for each participant across all remaining trials separately for congruent and incongruent trials, pooled across tone frequency. The raw correlation values were submitted to Fisher’s Z-transformation. The transformed correlations did not significantly differ between congruent (*M* = 0.85, *SD* = 0.40) and incongruent trials (*M* = 0.77, *SD* = 0.34), *t*_(18)_ = –1.68, *p* = .110, *d_z_* = 0.39, *BF*_10_ = 0.776. Neither did the mean absolute differences in AUC differ significantly between congruent and incongruent trials (congruent trials: *M* = 230 N*ms, *SD* = 220 N*ms; incongruent trials: *M* = 249 N*ms, *SD* = 288 N*ms), *t*_(18)_ = 0.99, *p* = .336, *d_z_* = 0.23, *BF*_10_ = 0.365.

Peak force returned similar results: No statistically significant differences were found between the Z-transformed correlations (Congruent: *M* = 0.86, *SD* = 0.38, Incongruent: *M* = 0.83, *SD* = 0.34); *t*_(18)_ = –0.82, *p* = .426, *d_z_* = 0.19, *BF*_10_ = 0.319, or the mean absolute force differences (Congruent: *M* = 1.29 N, *SD* = 0.93 N, Incongruent: *M* = 1.32 N, *SD* = 1.04 N), *t*_(18)_ = –0.74, *p* = .466, *d_z_* = 0.17, *BF*_10_ = 0.304.

As a complementary analysis, we compared the mean AUC values on the prime and probe, irrespective of condition, and found that responses to the probe were more forceful (*M* = 744 N*ms, *SD* = 694 N*ms) than actions eliciting the prime (*M* = 543 N*ms, *SD* = 652 N*ms, *t*_(18)_ = –3.23, *p* < .005, *d_z_* = 0.74, *BF*_10_ = 9.867). The difference was also present when peak forces were compared (Probe: *M* = 4.78 N, *SD* = 3.28 N; Prime: *M* = 3.58 N, *SD* = 3.09 N, *t*_(18)_ = –4.97, *p* < .001, *d_z_* = 1.14, *BF*_10_ = 283.815).

### Reaction time analysis

Reaction times were faster on congruent trials (*M* = 316 ms, *SD* = 120 ms) than on incongruent trials (*M* = 332 ms, *SD* = 107 ms), *t*_(18)_ = 2.08, *p* = .053, *d_z_* = 0.48, *BF*_10_ = 1.359.

### Pinch duration analyses

Post-hoc exploratory analyses of the corrected correlations between prime and probe pinch durations for congruent and incongruent trials were individually calculated, and submitted to paired *t*-tests. For the exploratory analyses we used Bonferroni adjusted alpha levels of .025 per test (.05/2). A significant difference between the correlations was found (*t*_(18)_ = 2.67, *p* = .016, *d_z_* = 0.61, *BF*_10_ = 3.617), with pinch durations being more similar on congruent (*M* = 0.75, *SD* = 0.33) than on incongruent trials (*M* = 0.63, *SD* = 0.27). The mean absolute pinch duration differences between prime and probe, however, did not differ significantly between congruent and incongruent trials (Congruent: *M* = 30 ms, *SD* = 23 ms, Incongruent: *M* = 34 ms, *SD* = 31 ms, *t*_(18)_ = 1.54, *p* = .140, *d_z_* = 0.35, *BF*_10_ = 0.652).

## Discussion

**Figure 2:**
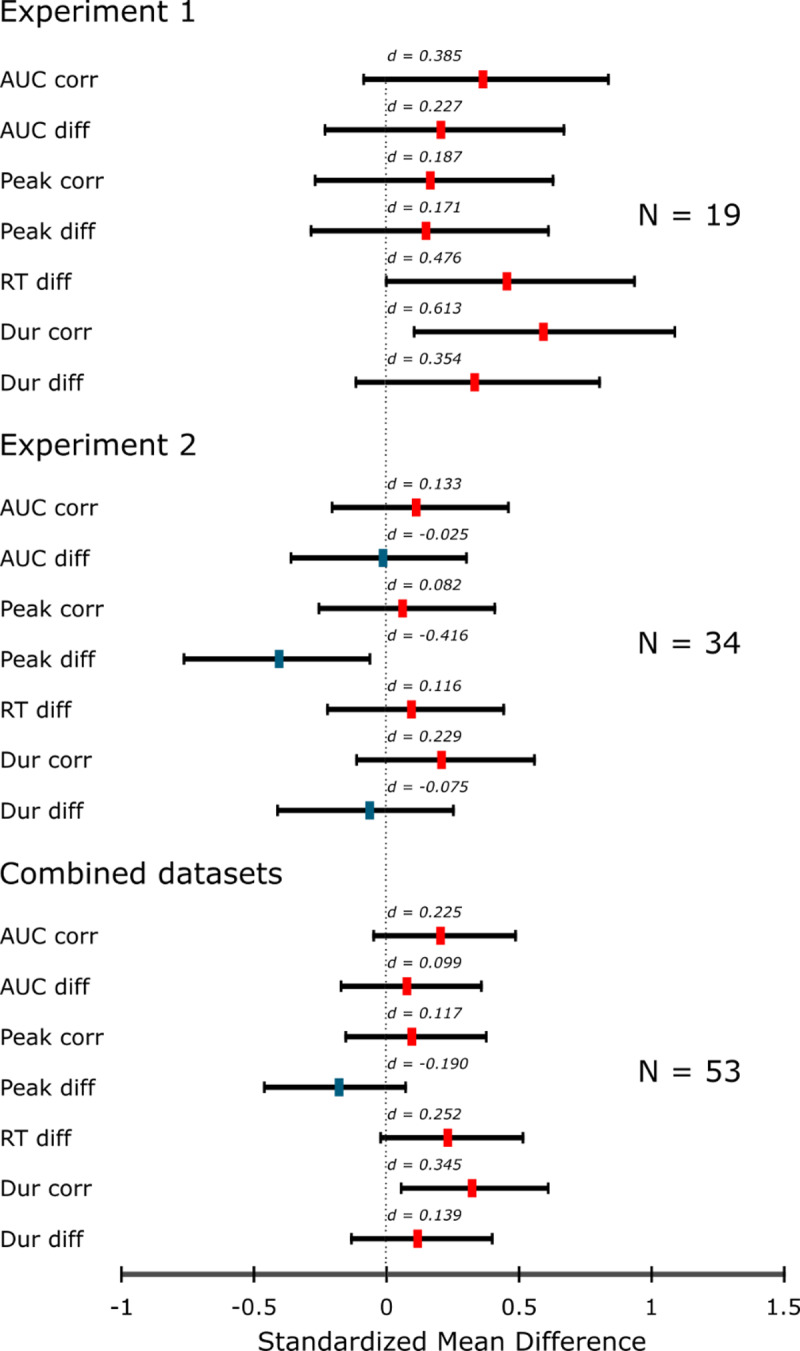
Effect sizes and corresponding 95% confidence intervals for each analysis presented in this study. *Corr* – Fisher-Z-transformed intraindividual correlation of prime and probe across trials between conditions. *Diff* – mean absolute difference of prime and probe between tone repetitions and tone switches. *RT diff* – reaction time difference between conditions. *Dur* – pinch duration.

In Experiment 1 we inspected a variety of parameters characterizing force application during pinching to investigate the presence of action-effect binding. The main hypothesis was that these parameters should be more similar when a previous response effect from a prime trial repeats rather than changes in a subsequent probe trial. A summary of the effect sizes is presented in the top part of Figure 2. Our main measures of interest were the temporal integral (AUC) of the applied force and peak force. Although in all four analyses the direction of the effect was compatible with the assumption that force application patterns might get bound to and retrieved by auditory stimuli, none of the comparisons reached the conventional level of statistical significance (*p* > .05). The post-hoc comparison of the correlation between prime and probe-related pinch durations showed that these were more similar in congruent than in incongruent trials. For the absolute difference measure, however, no significant difference was found. One plausible explanation for this difference in effect sizes is that in the case of absolute difference calculations we used the mean to characterize the participants’ action tendencies. Because this form of aggregation characterizes the data on a per participant level, it might not be able to grasp the variability present in the data to the same extent as correlations do (on a per-trial level).

The longer reaction time in incongruent trials is compatible with the notion that binding occurred. While in the literature we can see reaction time differences in the case of tone repetitions compared to alternations in choice tasks (Moeller et al., 2016), this result demonstrates that such reaction time differences can be found when only one response option is available.

It is also evident that self-initiated actions eliciting the prime effect were much softer than responses to the probe. This may be caused by differences in the initiation of spontaneous and responsive actions, and thus suggest that the arrangement might not be ideal for comparisons assessing similarity. Another factor that could have contributed to the force difference between the two actions was the relatively rare occurrence of catch trials. Because in 20% of the trials a second tone was absent, there could have been a strong anticipatory effect on the probe that was not present on the prime, further contributing to the dissimilarity between the two actions.

All-in-all, while the results were inconclusive regarding the presence of binding and retrieval of irrelevant dynamic action features, some of the results of Experiment 1 are compatible with the assumption that binding between the action and its irrelevant effect occurs. Nonetheless, their magnitude is much smaller than the influences observed in the case of discrete and/or task-relevant features reported in the literature, especially compared to response repetition rates (Dutzi & Hommel, 2009; Janczyk et al., 2012; Schwarz et al., 2019).

## Experiment 2

We conducted Experiment 2 to study the hypothesized effect with greater statistical power: we recorded data from more participants and adjusted certain aspects of the paradigm.

1) First, as the results of Experiment 1 show, pinches in response to the probe were stronger than the spontaneous pinches eliciting the prime. To mitigate this, an auditory go signal was introduced, and thus the action eliciting the prime was also a response to an auditory signal, just like the response to the probe. To avoid interference with the pitch manipulation, a brief white noise was used as the go signal.

2) The impact of potential anticipatory effects were reduced. The intertrial wait time was extended to a random interval between 3 and 4 seconds, thus making the go signal less predictable. Additionally, because in Experiment 1 some participants had relatively large error rates on the catch trials (which occurred on 20% of the trials), the number of catch trials was increased (to 50% of the trials).

3) Because in Experiment 1 many participants had reaction times under 250 ms, the duration of the tones was reduced to 150 ms (instead of 300 ms).

4) The final alteration was the introduction of a tone effect on the probe as well. Because the presence of on-the-fly adjustments to force (i.e. that later parts of the participants’ pinch are modulated by a reaction to the onset of the elicited sound, Cao et al., 2020) cannot be excluded, especially in the case of longer pinches, we reasoned that adding an action effect to the probe-related pinch would reduce potential force differences between prime and probe-related actions. This last tone was the high- or low-pitched tone selected randomly.

Because the procedure was very similar to Experiment 1, in the following Method section we only describe details that differed in Experiment 2.

Experiment 2 was preregistered at https://aspredicted.org/y5iz4.pdf.

## Method

### Participants

We aimed to detect an effect size of *d_z_* = 0.5 with 1-β = .80 and *α* = .05. To determine the required sample size, we ran a power analysis using the *pwr* package for R, version 1.3–0. Thus, our predetermined and final sample size was 34. Due to a deviation from the experimental protocol (the screen showing the force signal in the experimental chamber was not turned off, and the signal was visible for the participant during part of the experiment), data from one participant could not be included in the analyses, so we invited an additional participant to reach the target sample size. The final sample included 21 women and 13 men (*M*_age_ = 21 years, *SD* = 2 years, range = 18–27 years), who were mostly right-handed (5 participants were left-handed). No participant had taken part in Experiment 1.

### Stimuli, task and procedure

After the familiarization phase, the experiment started with the following instructions in Hungarian: *‘The task is to wait for a brief noise, then quickly pinch the device. The pinch will be followed by a sound, and sometimes by a second one as well. If you hear a second sound, pinch the device! The second pinch is always followed by another sound, but you do not have to react to this. Let me know if we can start.’*. As shown in Figure 2, an experimental trial started with an intertrial wait time of 3000–4000 ms, after which a 50 ms long (including 10 ms rise and 10 ms fall times), 10 kHz lowpass-filtered white noise was presented with an intensity of 35 dB,^2^ that served as a go signal for the prime. The 50 ms duration was chosen to prevent overlap with the following action-effect tone. If the participant pinched before the go signal (the white noise), the following text appeared in Hungarian: *‘Wait for the noise, please!’*, and the trial was repeated. The participant’s pinching response to the go signal elicited a 150 ms long, 440Hz-or 1175-Hz tone at 60 dB with 10 ms rise and fall times. 600 ms after the first pinch, a second tone was presented (pseudo-randomly) in half of the trials. The second tone could be congruent (i.e. repetition of the first tone) or incongruent (i.e. tone alternation). Half of the trials featuring a second tone were congruent, the other half incongruent – their order was pseudo-randomized. If the participant pinched within the 600 ms following the onset of the first tone, a warning appeared for 1 s: *‘Wait for the sound, please!’* The pinch was registered as a false alarm and the trial ended. The same text was displayed if the participant pinched on a catch trial. No tone effect was presented when the participant pinched (erroneously) on a catch trial. Pinching the FSR in response to the second tone onset elicited another tone effect. This again could be the high- or the low-pitched tone with 50% probability. If the participant did not pinch within 1200 ms after the onset of the second tone, the trial was recorded as a missed event. For catch trials, the total time until the end of the trial was 1800 ms (600 ms following the self-induced tone onset plus 1200 ms response interval).

**Figure 3:**
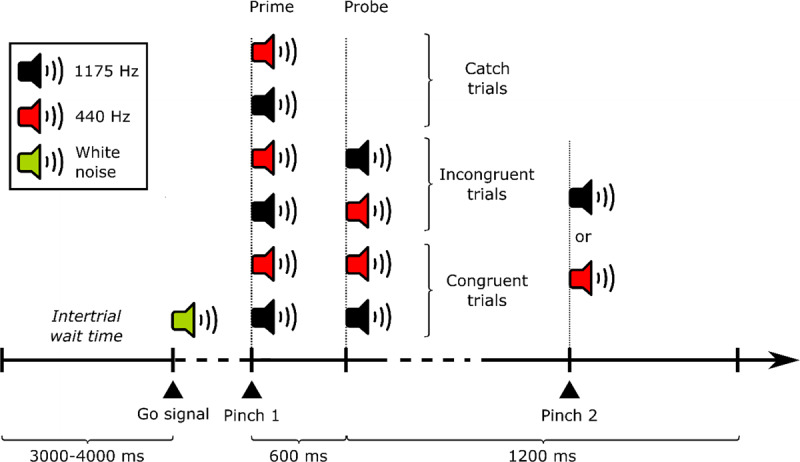
Trial structure in Experiment 2. The prime part of each trial featured a brief noise that served as a go signal for the first pinch that immediately triggered either a high or low effect tone. The probe part then either repeated this tone as a stimulus (congruent trials), featured the alternative stimulus (incongruent trial) or did not present any tone (catch trial). Participants were instructed to perform a second pinch whenever they heard a second tone but to refrain from responding in catch trials. The second pinch elicited another tone effect with the pitch (high or low) selected randomly with 50% probability. The timing represented on the horizontal axis is not to scale.

The experiment started with one practice block containing 20 trials (10 congruent, 10 incongruent and no catch trials) and was followed by 9 experimental blocks containing 40 trials each. Each experimental block consisted of 10 congruent trials, 10 incongruent trials and 20 catch trials (see Figure 3 for the trial procedure in Experiment 2).

#### Data selection and analyses

Trials with reaction times faster than 100 ms (whether on the prime or probe) were rejected (on average 0.1% of all trials, *SD* = 0.3%, range = 0.0–1.7%), along with late outliers with reaction times above 3 seconds^3^ (on average 0.2% of all trials, *SD* = 0.4%, range = 0.0–2.2%). We then further rejected trials with response times above a z-score of 2.5 (representing on average 3.9% of all trials per participant, *SD* = 1.2%, range = 2.2–7.8%). As in Experiment 1, trials with pinch durations equal to or above 600 ms on the prime (5.7% of the remaining trials, not including tone absent trials, *SD* = 8.3%, range = 0.0–27.0%) or probe (2.2% of the remaining trials, not including tone absent trials, *SD* = 4.0%, range = 0.0–18.0%) were rejected.^4^ After this rejection procedure, the average number of remaining trials per participant was 76 (*SD* = 11.2, range = 39–88) in the congruent condition, and 75 (*SD* = 10.7, range = 40–87) in the incongruent condition.

Additionally, trials with z-scores below or above 2.5 for any dependent variable were rejected separately from each analysis.

As in Experiment 1, peak force, the integral of force (AUC), and reaction times were used to characterize the actions. We again compared 1) the intraindividual correlation of prime and probe force across trials between conditions, 2) the mean absolute difference of prime and probe force between tone repetitions and tone switches and 3) reaction times on congruent and incongruent trials. We also performed the same exploratory analyses as in Experiment 1 and calculated Bayes Factors for the main analyses.

## Results

### Error rates and misses

The rate of misses (i.e. trials where the participant did not pinch on the probe) was low: on average 2.2% of all trials (*SD* = 3.0%, range = 0.0–9.4%). Similarly low was the number of premature pinches on the probe (i.e. pinching before the arrival of the sound): 0.3% of trials (*SD* = 0.7%, range = 0.0–3.3%). The mean number of trial restarts per participant was less than one (*M* = 0.2%, *SD* = 0.5%, range = 0.0–2.8%). False alarms (when the participant pinched on catch trials) were rare (*M* = 0.2%, *SD* = 0.6%, range = 0.0–3.1%). We did not reject any participant’s data because of these errors and misses.

### Force analyses

The corrected individual prime-probe correlation coefficients did not significantly differ between congruent and incongruent trials for the AUC (Congruent: *M* = 0.88, *SD* = 0.35; incongruent: *M* = 0.85, *SD* = 0.32, *t*_(33)_ = –0.78, *p* = .443, *d_z_* = 0.13, *BF*_10_ = 0.243). Similarly, comparisons of absolute prime and probe differences, showed no significant differences (AUC: Congruent: *M =* 208 N*ms, *SD* = 150; incongruent: *M* = 207 N*ms, *SD* = 150 N*ms; *t*_(33)_ = 0.143, *p* = .887, *d_z_* = 0.03, *BF*_10_ = 0.186).

Using peak force values for the corrected prime-probe correlation comparisons gave similar results (Congruent: *M* = 0.92, *SD* = 0.38; Incongruent: *M* = 0.9, *SD* = 0.36; *t*_(33)_ = –0.48, *p* = .634, *d_z_* = 0.08, *BF*_10_ = 0.205). However, mean absolute peak force differences between prime and probe were significantly larger in congruent (*M* = 0.93 N, *SD* = 0.56 N) than in incongruent trials (*M* = 0.84 N, *SD* = 0.43; *t*_(33)_ = –2.43, *p* = .021, *d_z_* = 0.42, *BF*_10_ = 2.329).

**Figure 4:**
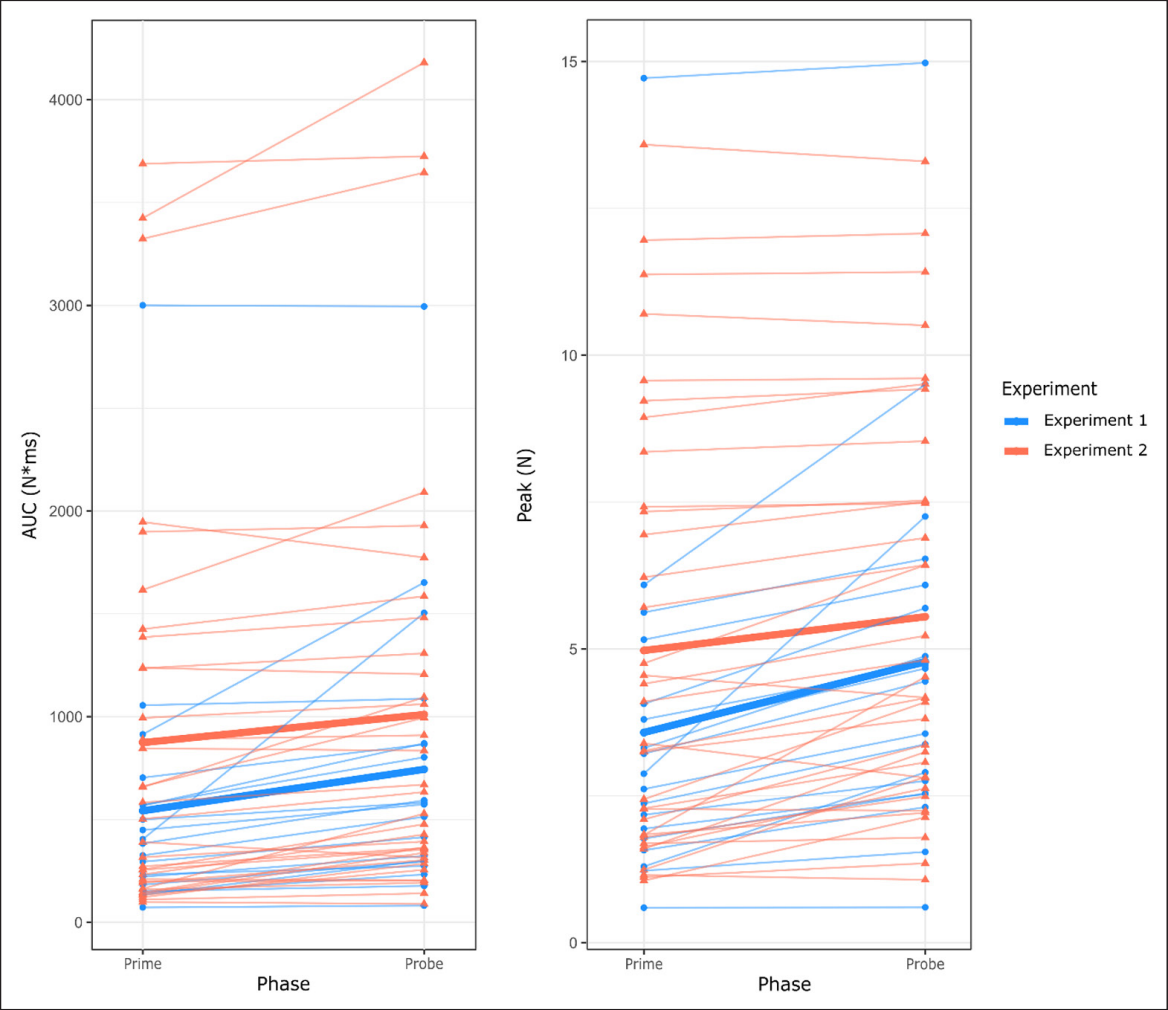
Force exertion in Experiments 1 and 2 using AUC (left) and peak force (right). Thin lines connect the data points of each participant, thick lines depict group-level averages.

We subsequently compared actions on the prime and probe, irrespective of the congruency of the trials. Pinches on the probe were carried out with significantly larger force exertion (AUC: *M* = 1011 N*ms, *SD* = 1053 N*ms; Peak force: *M* = 5.55 N, *SD =* 3.4 N) than pinches on the prime (AUC: *M* = 874 N*ms, *SD* = 987 N*ms, *t*_(33)_ = –4.43, *p* < .001, *d_z_* = 0.76, *BF*_10_ = 261.903; Peak force: *M* = 4.98 N, *SD =* 3.66 N, *t*_(33)_ = –4.74, *p* < .001, *d_z_* = 0.81, *BF*_10_ = 593.424). As Figure 4 illustrates, this difference between the two trial parts was similar to Experiment 1 when using AUC (*F*_(1,51)_ = 1.33, *p* = .255, η^2^*_G_* = .25), with the interaction between experiment (Exp. 1 or 2) and part (prime or probe) being non-significant (*F*_(1,51)_ = 1.10, *p* = .300, η^2^*_G_* < .01. When it comes to peak force, the main effect of experiment was similarly non-significant, (*F*_(1,51)_ = 1.25, *p* = .269, η^2^*_G_* = .02), but there was a small but significant interaction with part (*F*_(1,51)_ = 6.75, *p* = .012, η^2^*_G_* < .01).

### Reaction time analysis

Reaction times to probes did not significantly differ between congruent (*M* = 368 ms, *SD* = 84 ms) and incongruent (*M* = 371 ms, *SD* = 85 ms, *t*_(33)_ = 0.67, *p* = .505, *d_z_* = 0.12, *BF*_10_ = 0.227) trials.

### Pinch duration analyses

For the pinch duration analyses we again used Bonferroni adjusted alpha levels of .025 per test (.05/2). The corrected correlations between prime and probe pinch durations did not significantly differ between congruent (*M* = 0.74, *SD* = 0.26) and incongruent trials (*M* = 0.68, *SD* = 0.27; *t*_(33)_ = –1.34, *p* = .191, *d_z_* = 0.23, *BF*_10_ = 0.414). Neither did the mean absolute prime and probe pinch duration differences (congruent trials: *M* = 30 ms, *SD* = 20 ms; incongruent trials: *M* = 30 ms, *SD* = 18 ms; *t*_(33)_ = –0.44, *p* = .666, *d_z_* = 0.08, *BF*_10_ = 0.201).

## Discussion

In Experiment 2 we intended to expand the results of Experiment 1 and to study the hypothesized effects with greater statistical power. We refined some aspects of the paradigm with a focus on improving the similarity of the two parts (prime and probe) and the reduction of possible interferences.

The results of Experiment 2 converged with the results of Experiment 1, indicating null effects, and overall smaller effect sizes (see Figure 2, middle). The only significant difference was in the mean absolute peak force difference between prime and probe, with a larger absolute difference in congruent trials. In light of the other results (especially the correlation comparisons of Experiments 1 and 2 and the results of the combined datasets, see below), and the caveat described in the discussion of Experiment 1, this result warrants a cautious interpretation.

The large difference between the two pinches (on the prime and probe) indicate that the addition of a go signal (the white noise) and the tone effect on the probe did not increase the similarity of the two pinches.

## Pooled analyses

In a final analysis, we combined the datasets of the two experiments to increase statistical power and performed the same comparisons on the pooled data (*N* = 53).

### Force analyses

The corrected individual prime-probe correlation coefficients did not differ significantly when using AUC, between congruent (*M* = 0.87, *SD* = 0.37) and incongruent trials (*M* = 0.82, *SD* = 0.33, *t*_(52)_ = –1.64, *p* = .107, *d_z_* = 0.23, *BF*_10_ = 0.524). No significant difference was found for the mean absolute differences either (congruent trials: *M* = 216 N*ms, *SD* = 176 N*ms, incongruent trials: *M* = 222 N*ms, *SD* = 208 N*ms; *t*_(52)_ = 0.72, *p* = .476, *d_z_* = 0.10, *BF*_10_ = 0.191).

The analysis using peak force gave similar results in the case of corrected correlation comparisons (congruent trials: *M* = 0.90, *SD* = 0.38; incongruent trials: *M* = 0.87, *SD* = 0.35; *t*_(52)_ = –0.85, *p* = .398, *d_z_* = 0.12, *BF*_10_ = 0.211) and mean absolute differences between prime and probe (Congruent: *M* = 1.06 N, *SD* = 0.73 N; Incongruent: *M* = 1.02 N, *SD* = 0.74 N; *t*_(52)_ = –1.39, *p* = .172, *d_z_* = 0.19, *BF*_10_ = 0.368).

### Reaction time analysis

The difference in reaction times was not significant, with pinches being slightly faster on congruent (*M* = 349 ms, *SD* = 100 ms) than incongruent trials (*M* = 357 ms, *SD* = 94 ms, *t*_(52)_ = 1.84, *p* = .072, *d_z_* = 0.25, *BF*_10_ = 0.714).

### Pinch duration analyses

We used adjusted alpha levels of .025 per test (.05/2). The corrected correlations between prime and probe pinch durations were higher in congruent (*M* = 0.75, *SD* = 0.28) than in incongruent trials (*M* = 0.67, *SD* = 0.27, *t*_(52)_ = 2.51, *p* = .015, *d_z_* = 0.35, *BF*_10_ = 2.553). For mean absolute differences, however, no significant difference was found (congruent trials: *M* = 30 ms, *SD* = 21; incongruent trials: *M* = 0.31 ms, *SD* = 0.23 ms; *t*_(52)_ = 1.01, *p* = .316, *d_z_* = 0.14, *BF*_10_ = 0.243).

## Discussion

The combined analyses of the two datasets reflected the results presented separately in Experiments 1 and 2 (see Figure 2, bottom). There was a tendency for actions in congruent trials to be more similar to each other when compared to incongruent trials. This was most apparent in correlation comparisons, especially in the case of pinch durations. However, as mentioned earlier, the effect sizes in these comparisons were much smaller than the ones reported in the literature. Even the sample size provided by the combined datasets amounts to only 57% statistical power to detect an effect size of *d_z_* = 0.30; this effect size is considerably smaller than what is commonly found in the literature, where responses are defined in terms of their task-relevant, categorical action features. Thus, considering that most comparisons yielded null effects, we hesitate to draw strong conclusions from these results, but we believe that they pave the way for assessing the question of binding and retrieval for task-irrelevant action features much more efficiently in future research.

## General Discussion

In the present study we focused on task-irrelevant, metric properties of actions and investigated action-effect bindings for these features in two experiments. Using a modified prime-probe design involving a single response option, pinching, we assessed whether different features of such a quick, ballistic action get bound to environment-related sensory action effects and whether they are retrieved by the repetition of these stimuli. As summarized in Figure 2, the results were heterogeneous. Force application patterns characterized by AUC and peak measurements showed only one significant difference in Exp. 2 and that in the direction opposite to the hypothesis: the prime-probe mean absolute peak force differences were larger in the congruent condition, that is when the tone was repeated. Reaction times to the probe were faster when the tone was repeated in Exp. 1, which is compatible with results observed in choice reaction tasks (e.g., Moeller et al., 2016). Our explorative comparisons of pinch durations showed significantly higher prime-probe correlations in congruent trials in Exp. 1 and in the pooled analysis, which is also compatible with the hypothesis that task-irrelevant pinch duration may be bound to and retrieved by the repetition of the tone.

We used several measures to characterize the (dis)similarity of closely following actions, and these measures may have different sensitivities. First, effect sizes for the comparisons between prime-probe force correlations were larger in both experiments than for comparisons of mean absolute differences. As suggested above, correlation may better capture the trial-level variability than mean absolute differences do. Second, AUC seemed to be a more sensitive indicator than peak force, which might be because AUC is calculated from all the available sampling points, and thus may provide a better signal-to-noise ratio than peak force.

Besides differences in sensitivity (signal-to-noise ratio), these measures may also capture 1) different aspects of the actions; and they may also 2) imperfectly capture the features that are naturally used by the cognitive system to represent the actions. In the Introduction, we alluded to the idea that kinematic features of complex movements are *readily observable*. When a participant produces a reaching movement, she can monitor the action by relying on both proprioceptive information from the moving limb as well as seeing the ongoing action itself, coupled with predictions about the position of the arm using information from previous motor commands (Shadmehr et al., 2010). The different sources of information, be it open-loop or closed-loop feedback (Adams, 1971; Wolpert et al., 2011), can be weighted and combined according to their reliability (Kalman, 1960). We can go a step further and speculate that in the case of movements toward visual targets, when available, the participant might rely more on the visually perceived kinematic information (position, velocity) than on proprioception to produce the desired movement (though these are both relevant in different stages of movement planning, Sarlegna & Sainburg, 2009). Consequently, some aspects of an action may disproportionately dominate a representation because of *intentional weighting* (Memelink & Hommel, 2013). In line with this idea, in our experiments, duration might be a feature that is weighted stronger than peak force, or the integral of the force, because it may be more readily observable, or because it may correspond better to the features the cognitive system relies on to represent the actions. Additionally, because the production of quick, brief pinches was part of the task instruction, this could have made action duration more task-relevant than action force. Whether or not there is a difference in intentional weighting when it comes to action force and action duration, both movement features are nominally task-irrelevant, non-categorical and highly correlated, and could thus be bound and retrieved, but possibly not to the same extent.

Other explanations relating to this difference may also be conceivable, such as a difference in retrieval (while action force might similarly get bound to sensory action effects, because of its irrelevance in this experimental setting, it might be retrieved to a lesser degree than action duration), or the different levels of noise in the two measurements. While future experiments are required to confidently answer these questions and to differentiate between the possible explanations, in the following, we discuss some aspects regarding the experimental design used in this study.

The results observed in our experiments might have been influenced by the inherent noise in our paradigm. As described above, the self-initiated actions in Experiment 1 tended to be much softer than the forced reactions on the probe, and the inclusion of a go signal in Experiment 2 did not markedly reduce this observable difference. Because action force was not a task-relevant feature, coupled with the briefness of actions, the overall variability of actions might have also been low. Future experiments could improve this design aspect by including a larger array of actions (possibly including softer and stronger actions). Implementing such changes would also address potential alternative explanations in terms of limited binding and retrieval effects for mere detection responses in the probe (Huffman et al., 2018, Schöpper et al., 2020; note that participants did not have to process tone identity to decide whether they should respond or not).

An additional point worth noting with regards to the experimental design used in this study is the fact that participants held the FSR between their index finger and the thumb, which periodically could have resulted in slight shifts in the way they initiated the actions. While it is unlikely to significantly impact the results, this might be a further form of inherent noise that could be minimized in future experiments (e.g., by taping the FSR to the surface of a table).

The different options for optimizing the present design promise to increase effect sizes across measures consistently. Albeit being a step in the right direction, such gains should not be expected to be overly strong, however, and the true effect size might indeed be small. Evidence for this speculation comes from a related study that used a substantially different design to tackle the question of binding and retrieval for metric action features (Pfister et al., 2022). Participants in this study performed keypress responses to target letters, with two targets mapped on each of two responses. Such a design enables testing whether repeating a target stimulus, and thus the response, would retrieve the previous movement as assessed via response durations. This was indeed the case with response durations of two successive trials being more similar for target repetitions relative to response repetitions in the face of changed targets. The observed effects were decidedly small, however. Of note, this study only assessed similarity via differences in response durations and obtained an effect size of *d_z_* = 0.34 for this measure, which we only observed for correlations in the present design. Follow-up analyses suggested that applying the present correlation measure to this data produced a similar effect. Differences in response modality – keypress versus pinch responses – might be responsible for the different sensitivity of either correlation or difference measures so that future work would be well advised to use both ways of quantifying similarity of any movement parameter.

All-in-all, as depicted in Figure 2, the results of the performed analyses seem to point to the same general direction: repeating an auditory action effect on the probe tends to ensue in a response that is initiated faster and is more similar to the first action in different task-irrelevant action parameters. However, these effects are surprisingly small, hinting at the possibility that task-irrelevant action features might not be part of the representations and/or retrieved to the same extent as task-relevant features. While they are inconclusive, we believe that these results provide a stepping stone for designing more powerful experiments so that future work can tailor the experimental design to the distinctive properties of task-irrelevant, metric features that define actual body movements.

## Data Accessibility Statement

Data and analysis code are available on the Open Science Framework: https://osf.io/5vjg3.

## Footnotes

The analyses of response duration were partly motivated by a separate study also published in this Special Issue (Pfister et al., 2022). This study found small effects for binding and retrieval of response durations for keypress responses, mirroring the picture of overall small effects as observed in the present experiments.

The intensity of the go signal was selected so that simple reaction times would be largely similar for the three sounds, by running an informal pilot experiment, in which simple reaction times were recorded to the pure tones used in the experiment and white noise sounds of various intensities.

This added step of removing trials with reaction times longer than 3 s is a deviation from our preregistered procedure, however, we did not anticipate that on some occasions participants would be unsure whether or not they heard a noise (on the prime) or a tone (on the probe) and thus waiting and producing artificial reaction times – in some cases up to several seconds.

Removing trials with action durations above 600 ms is also an additional step not preregistered. As described in Exp. 1, our post-hoc reasoning was that in trials with such long actions on the prime, the onset of the second tone arrives before the first action ends.
